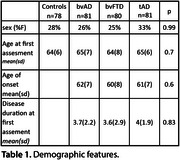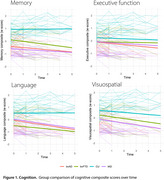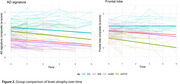# Cognitive and neuroimaging trajectories in the behavioral variant of Alzheimer's disease

**DOI:** 10.1002/alz70857_106431

**Published:** 2025-12-26

**Authors:** Ismael Luis Calandri, Jeffrey S Phillips, Pontus Tideman, Ellen Hanna Singleton, Renaud La Joie, Wiesje M. van der Flier, Laura E. Jonkman, Oskar Hansson, Gil D. Rabinovici, Yolande A.L. Pijnenburg, Rik Ossenkoppele, Sophie E. Mastenbroek

**Affiliations:** ^1^ Alzheimer center, VUMC, Amsterdam, Netherlands; ^2^ Penn Frontotemporal Degeneration Center, Department of Neurology, Perelman School of Medicine, University of Pennsylvania, Philadelphia, PA, USA; ^3^ Clinical Memory Research Unit, Department of Clinical Sciences Malmö, Faculty of Medicine, Lund University, Lund, Sweden; ^4^ Department of Psychiatry and Neurochemistry, University of Gothenburg, Mölndal, Västra Götalands län, Sweden; ^5^ Memory and Aging Center, Weill Institute for Neurosciences, University of California San Francisco, San Francisco, CA, USA; ^6^ Alzheimer Center Amsterdam, Neurology, Vrije Universiteit Amsterdam, Amsterdam UMC location VUmc, Amsterdam, Netherlands; ^7^ Amsterdam UMC, location VUmc, Department of Anatomy and Neurosciences, Section Clinical Neuroanatomy and Biobanking, Amsterdam, Netherlands; ^8^ Clinical Memory Research Unit, Department of Clinical Sciences Malmö, Lund University, Lund, Sweden; ^9^ UCSF Alzheimer's Disease Research Center, San Francisco, CA, USA; ^10^ Alzheimer Center Amsterdam, Neurology, Vrije Universiteit Amsterdam, Amsterdam UMC location VUmc, Amsterdam, Amsterdam, Netherlands; ^11^ Clinical Memory Research Unit, Department of Clinical Sciences, Lund University, Lund, Sweden

## Abstract

**Background:**

The behavioral variant of Alzheimer's disease (bvAD) is a rare atypical presentationcharacterized by early and prominent behavioral changes, clinically akin to the behavioral variant of fronto‐temporal dementia (bvFTD). The natural history of bvAD is poorly understood. This study investigates the progression of bvAD in a multinational cohort, comparing bvAD with matched bvFTD and typical AD (tAD) groups.

**Method:**

We analyzed 81 bvAD participants from four centers and matched them by age, sex, and education to bvFTD (*n* = 80), tAD (*n* = 81), and controls (*n* = 78). Participants completed longitudinal clinical assessments and underwent repeated structural MRI. We combined neurocognitive variables into domain‐specific composites. Furthermore, we extracted cortical thickness and volumetric MRI data using FreeSurfer andcomputed atemporal AD‐signature and a frontal region‐of‐interest. Linear mixed models were used to evaluate cognitive and neuroimaging trajectories. The model coefficients are presented as standardized (β), and the effect is assessed through estimated marginal means (EMM).

**Result:**

Demographic features are shown in Table 1. Subjects with bvAD exhibited significant decline in executive function (β=‐0.62, 95%CI[‐1.02, ‐0.22], *p* = 0.03, EMM=‐0.19), memory (β=‐1.62, 95%CI[‐1.90, ‐1.33], *p* < 0.001, EMM=‐0.49), language (β=‐0.95, 95%CI[‐1.31, ‐0.59], *p* < 0.01, EMM=‐0.18), and visuospatial function (β=‐0.80, 95%CI[‐1.31, ‐0.28], *p* < 0.05) compared to controls. Compared to bvFTD, individuals with bvAD showed relatively greater memory (*p* = 0.005) and language (*p* = 0.04) preservation over time, while no significant differences were observed in visuospatial function (*p* = 0.65) or executive function (*p* = 0.4). In contrast, bvAD did not differ significantly from tAD in memory (*p* = 0.1), language (*p* = 0.6), visuospatial function (*p* = 0.99), or executive function.

Individuals with bvAD exhibited significant decline in the AD‐signature (β=‐0.91, 95%CI[‐1.34, ‐0.48], EMM=‐0.49) and frontal ((β=‐0.66, 95%CI[‐1.16, ‐0.15], EMM=‐0.29) regions‐of‐interest compared to controls. Compared to tAD, no significant differences were found in both regions of interest (*p* = 0.61, *p* = 0.91). Compared to bvFTD, individuals with bvAD showed significantly greater atrophy in the AD‐signature regions (*p* = 0.02) and significantly less atrophy in frontal lobe (*p* = 0.03).

**Conclusion:**

The progression of bvAD differs both cognitivelyand anatomically from bvFTD, while showing a progression pattern that is very similar to tAD. These results underscore the importance of investigating AD pathology in the context of cognitive‐behavioral decline.